# Directed evolution of an *E. coli* inner membrane transporter for improved efflux of biofuel molecules

**DOI:** 10.1186/1754-6834-6-81

**Published:** 2013-05-21

**Authors:** Jee Loon Foo, Susanna Su Jan Leong

**Affiliations:** 1 School of Chemical and Biomedical Engineering, Nanyang Technological University, 62 Nanyang Drive, Singapore 637459, Singapore

**Keywords:** Protein engineering, Synthetic biology, Efflux, Biofuel, Directed evolution, Transporter

## Abstract

**Background:**

The depletion of fossil fuels and the rising need to meet global energy demands have led to a growing interest in microbial biofuel synthesis, particularly in *Escherichia coli,* due to its tractable characteristics. Besides engineering more efficient metabolic pathways for synthesizing biofuels, efforts to improve production yield by engineering efflux systems to overcome toxicity problems is also crucial. This study aims to enhance hydrocarbon efflux capability in *E. coli* by engineering a native inner membrane transporter, AcrB, using the directed evolution approach.

**Results:**

We developed a selection platform based on competitive growth using a toxic substrate surrogate, which allowed rapid selection of AcrB variants showing enhanced efflux of linear and cyclic fuel molecule candidates, *n-*octane and α-pinene. Two mutants exhibiting increased efflux efficiency for *n*-octane and α-pinene by up to 47% and 400%, respectively, were isolated. Single-site mutants based on the mutations found in the isolated variants were synthesized and the amino acid substitutions N189H, T678S, Q737L and M844L were identified to have conferred improvement in efflux efficiency. The locations of beneficial mutations in AcrB suggest their contributions in widening the substrate channel, altering the dynamics of substrate efflux and promoting the assembly of AcrB with the outer membrane channel protein TolC for more efficient substrate export. It is interesting to note that three of the four beneficial mutations were located relatively distant from the known substrate channels, thus exemplifying the advantage of directed evolution over rational design.

**Conclusions:**

Using directed evolution, we have isolated AcrB mutants with improved efflux efficiency for *n*-octane and α-pinene. The utilization of such optimized native efflux pumps will increase productivity of biofuels synthesis and alleviate toxicity and difficulties in production scale-up in current microbial platforms.

## Background

Worldwide industrialization has resulted in an increasing global demand for energy, particularly fossil fuel. To meet the growing energy needs and tackle escalating environmental concerns, much effort has been directed towards biosynthesis of biofuels from renewable sources to substitute petroleum-based fuels [1-6]. Although bioethanol synthesis from biorenewable feedstocks formed the earliest success story in the biofuel arena, its low energy density and hygroscopicity led to efforts to produce longer chain hydrocarbons with higher energy density and lower hygroscopicity.

Many hydrocarbons are, however, toxic to microorganisms and endogenous biosynthesis of these compounds is expected to have adverse effects on cell growth and production yield [7]. One of the ways microorganisms mitigate the deleterious effect of toxic compounds is to employ efflux pumps to expel noxious molecules, hence enhancing cell survival [8]. While cell viability may not be a major concern for production of less toxic biofuels such as medium and long chain alkanes [8], these efflux pumps can help to accelerate release of the hydrocarbons into the growth media for harvest. Thus, utilizing efflux pumps to complement biosynthesis of biofuel candidates can be extremely beneficial for improving production yield.

Dunlop et al. [9] recently discovered novel bacteria efflux pumps that could be expressed heterologously in *E. coli* to improve hydrocarbon tolerance. Although it may seem straightforward to increase extrusion of biofuel molecules by over-expressing the efflux pumps, this could compromise cell viability, which is undesirable [10]. A more favorable approach is to improve the pump efficiency for secreting hydrocarbons. Alternatively, efflux pump systems native to *E. coli* can be optimized for enhanced efflux efficiency by protein engineering. The latter strategy minimizes problems with protein aggregation and inactivity that may be associated with heterologous expression of foreign proteins [11,12], which forms the basis of this study.

We chose to study the well-characterized AcrAB-TolC efflux pump system, which consists of an inner membrane transporter AcrB and an outer membrane channel TolC held together by a periplasmic membrane fusion protein AcrA [13]. It has a remarkably wide substrate specificity, including antibiotics, dyes, detergents, as well as many solvent molecules [13]. Since AcrB plays a critical role in substrate recognition and transport in the tripartite system [14], we endeavored to engineer this protein to improve the efflux efficiency of the pump system, particularly to accelerate the exportation of biofuel molecules into the growth medium for easy recovery. The target molecule chosen for this study is *n-*octane, a potential fuel substitute [5,15]. To this end, we employed directed evolution to screen for AcrB variants with improved *n-*octane efflux rate. Unlike fluorescent substrates like Nile Red [16], *n-*octane has no chromophore, thus efflux rate of *n-*octane from *E. coli* cannot be conveniently determined spectroscopically in real time. *n-*Octane is also chemically inert and difficult to derivatize for colorimetric analysis. In the absence of a high-throughput analytical method, we sought to increase the screening throughput by competitive growth selection of AcrB mutant pools. Based on the hypothesis that solvent resistance correlates to increased growth and efflux rate, subjecting *E. coli* cells expressing a mixture of AcrB variants to the toxicity of *n-*octane would enable the superior mutants to dominate the population, thus enriching the culture with AcrB mutants with improved efflux efficiency. Owing to the mild toxicity of *n-*octane to *E. coli*[8], growth selection using 1-octanol as a more toxic substrate surrogate [17] to exert stronger selection pressure was also subsequently investigated. Efflux rates of *n-*octane were determined to select for variants with enhancement in efflux efficiency. Finally, we studied the locations of the mutations found in the selected variants based on the reported crystal structure of AcrB (PDB ID: 2DHH) to rationalize the possible roles that the amino acid substitutions play in improving efflux of small biofuel molecules.

## Results and discussion

### Competitive growth assay for establishing conditions for library enrichment

**Figure 1:**
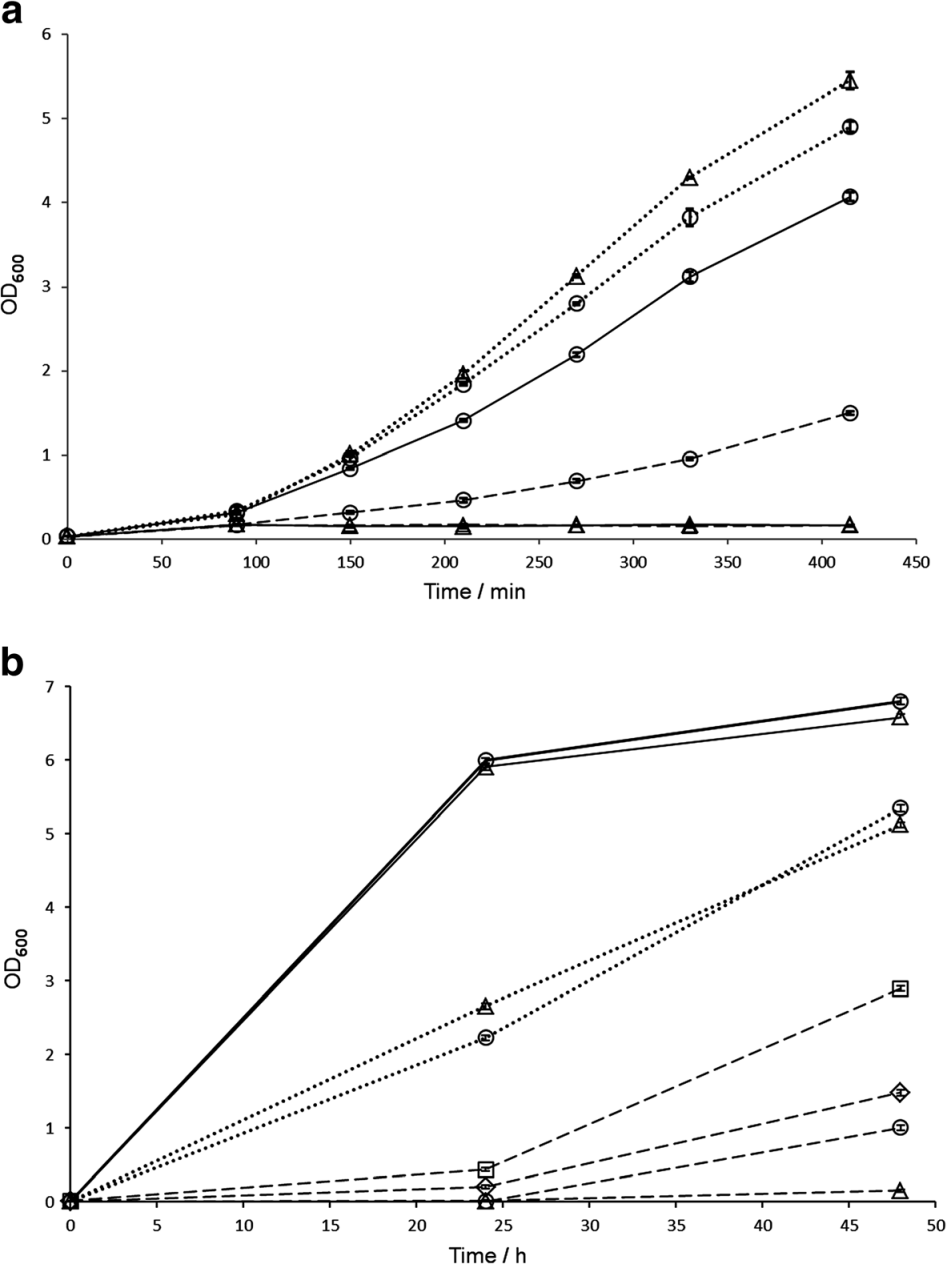
**Effects of (a)*n-*octane and (b) 1-octanol on the growth rates of JA300A/pMW119 (∆) and JA300A/pAcrB (○). **In (**a**), cell growth in the absence of *n-*octane are represented by dotted lines. Dashed lines depict cell growth in the presence of *n-*octane without IPTG induction. Solid lines illustrate the growth of IPTG-induced cells in the presence of *n-*octane. In (**b**), all cells were induced with IPTG. Cell growth in the presence of 0, 0.025 and 0.050% 1-octanol are shown in solid, dotted and dashed lines, respectively. Growth of mutants 1G1 (□) and 1G2 (◊) in 0.050% 1-octanol are overlaid.

To identify AcrB variants with improved *n*-octane efflux efficiency, we first set out to establish suitable conditions for growth selection by determining the hydrocarbon tolerance of *E. coli* K-12. To achieve this aim, the *E. coli* K-12 JA300, which has been used in several studies in solvent resistance [8,18-20], was manipulated to generate the *acrB*-inactivated derivative JA300A. The *acrB* gene was cloned into the low copy number plasmid pMW119 to create pAcrB. JA300A was transformed with (i) pAcrB (for plasmid complementation) and (ii) pMW119 (for a non-AcrB-expressing negative control strain) and examined for the inhibitory effects of *n-*octane and 1-octanol on the growth rates (Figure 1). In the absence of *n-*octane, JA300A/pMW119 and JA300A/pAcrB grew at very similar rates (Figure 1a). Addition of *n-*octane almost fully inhibited the growth of JA300A/pMW119 in the biphasic culture system saturated with *n-*octane and significantly retarded the proliferation of JA300A/pAcrB. Supplementation of the growth media with 0.1 mM isopropyl-β-D-thiogalactopyranoside (IPTG) to induce AcrB expression markedly increased the cell growth of JA300A/pAcrB to nearly the rate of the strains when *n-*octane was absent, but had no effect on JA300A/pMW119 (Figure 1a). The addition of IPTG neither enhanced nor inhibited growth rates of JA300A/pMW119 and JA300A/pAcrB in the absence of *n-*octane (Additional file 1: Figure S1). We therefore established that competitive growth enrichment of the mutant libraries with *n-*octane would be performed at basal expression level. Under this condition, the growth rate of cells with wild-type AcrB *n*-octane efflux efficiency is sufficiently lower than the maximum growth rate achievable by the cells. Thus, this condition applied adequate selection pressure to enrich the culture with cells possessing higher *n-*octane tolerance.

Competitive growth assays with 1-octanol were performed with varying concentrations of the substrate in growth media supplemented with 0.1 mM IPTG (Figure 1b). The difference in growth rates of JA300A/pMW119 and JA300A/pAcrB was insignificant in the presence of 0.025% 1-octanol, although growth was markedly inhibited for both compared to that without 1-octanol. When the 1-octanol concentration was further increased to 0.050%, the growth of JA300A/pMW119 was more significantly inhibited than JA300A/pAcrB, with a distinct difference in growth rate apparent between the two strains. Thus, competitive growth enrichment of the mutant libraries with 1-octanol was performed using induced cells with 0.050% of the substrate.

**Figure 2:**
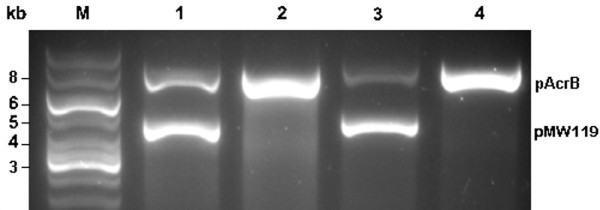
**Plasmids isolated from a mixture of JA300A/pMW119 and JA300A/pAcrB that have been subjected to selection conditions. **AcrB was expressed at basal level in lanes 1 and 2 while IPTG was used to induce protein expression in lanes 3 and 4. Lane 2 was subjected to growth selection with *n-*octane and lane 4 with 1-octanol. M denotes marker. The isolated plasmids were linearized with BamHI and the sizes correspond to those of pAcrB (7.4 kb) and pMW119 (4.2 kb).

The ability of the chosen selection condition to allow cells with higher hydrocarbon tolerance to dominate the population in the culture was tested. JA300A/pMW119 and JA300A/pAcrB grown separately were mixed in equal amounts and the diluted mixture was subjected to the selection conditions for growth. Cultures with no hydrocarbons were grown in parallel as controls. Plasmids isolated from the control cultures consisted of both pMW119 and pAcrB while those extracted from cultures grown under the selection pressure of the hydrocarbons contained only pAcrB (Figure 2). The elimination of non-AcrB-expressing JA300A/pMW119 under the selection conditions illustrates the successful enrichment of the cultures with JA300A/pAcrB, which possess higher tolerance to the hydrocarbons. These competitive growth assay results demonstrate the potential of selecting variants with improved hydrocarbon tolerance and possibly increased efflux efficiency from an AcrB mutant library.

### Efflux rate determination of *n*-octane

**Figure 3:**
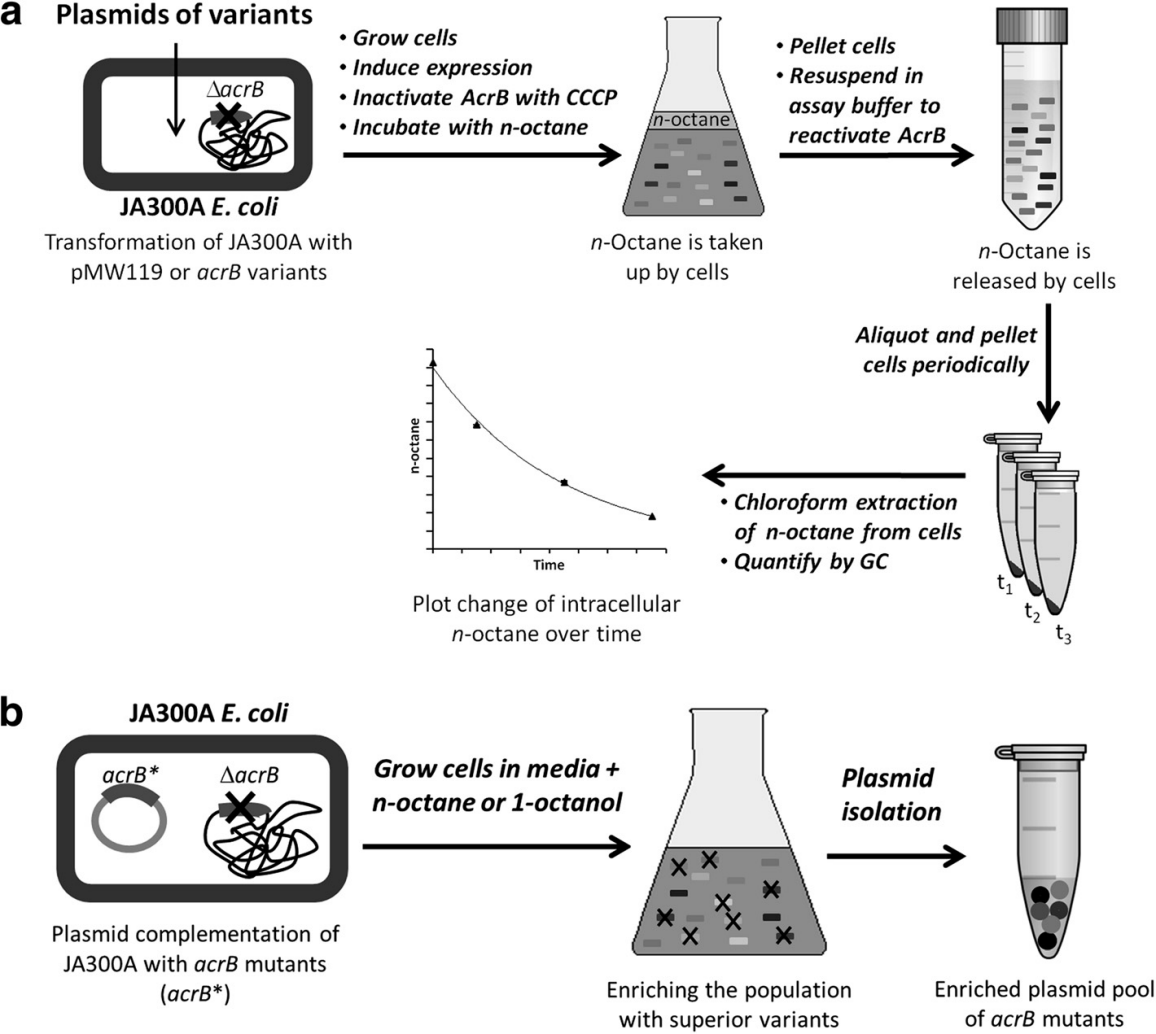
**Schematic diagram demonstrating the protocols for efflux assay and competitive growth enrichment. **(**a**) Efflux assay was performed in the *acrB*-inactivated *E. coli *strain JA300A transformed with the plasmids of interest. Expressed AcrB were inactivated to allow substantial uptake of *n-*octane. Resuspension of the cells in assay buffer containing glucose reactivated the cells and the change in intracellular *n-*octane content was quantified by GC-MS to determine the efflux rate. Discrete clones were isolated from libraries with improved AcrB mutants. These mutants were assayed using the same method except the cells were cultivated from single colonies. (**b**) JA300A was transformed with plasmids harboring *acrB* mutants. The cultures containing cells expressing a mixture of AcrB variants were grown in the presence of *n-*octane or 1-octanol. As inferior mutants were killed by the hydrocarbons, the population became enriched with superior AcrB that conferred increased tolerance. The plasmid mixtures were isolated after enrichment for efflux assay.

**Figure 4:**
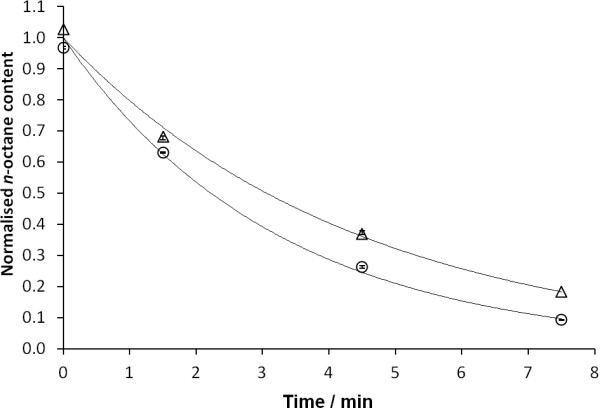
**Change of intracellular*n-*octane with time in JA300A/pMW119 (∆) and JA300A/pAcrB (○).** Intracellular *n-*octane content has been normalized to the respective initial mass in mg.

Owing to the chemical inertness of *n*-octane and a lack of chromophore, a high-throughput method for quantifying *n*-octane was not available. Therefore, in this study, the *n-*octane efflux rate was determined by a discontinuous assay using gas chromatography to quantify the intracellular *n*-octane over time by adaptation of reported protocols [8,16] (Figure 3a). JA300A/pMW119 and JA300A/pAcrB were cultivated and induced with IPTG for protein expression. AcrB, a proton-motive efflux pump, was inactivated by the ionophore carbonyl cyanide 3-chlorophenylhydrazone (CCCP) before *n-*octane was added to the cells to form a biphasic mixture saturated with *n*-octane. After incubating the cells with *n-*octane for uptake, the cells were resuspended in a buffer with glucose to reactivate AcrB. The cells were pelleted at regular time intervals and subjected to chloroform extraction to recover intracellular *n*-octane, which was subsequently quantified by gas chromatography analyses. The change in intracellular *n-*octane content over time was curve-fitted as a first-order process to estimate the rate constants for *n-*octane release (Figure 4) [8]. The efflux rate of *n-*octane by AcrB was calculated by taking the difference between the *n-*octane release rate constants of JA300A/pMW119 (expressing no AcrB) and JA300A/pAcrB (expressing wild-type AcrB) (Table 1).

**Table 1:** **Rates of*n-*octane and α-pinene efflux from JA300A expressing AcrB variants**

**Hydrocarbons**	**AcrB variants**	**Hydrocarbon release rate constant/min**^**-1**^	**AcrB hydrocarbon efflux rate constant**^**a**^**/min**^**-1**^	**Relative AcrB efflux rate**
*n*-octane	none	0.227 ± 0.007	-	-
	Wild-type	0.312 ± 0.010	0.085	1.00
	1G1	0.352 ± 0.011	0.125	1.47
	1G2	0.349 ± 0.009	0.122	1.44
	N189H	0.318 ± 0.018	0.091	1.07
	L357Q	0.290 ± 0.011	0.063	0.74
	T678S	0.318 ± 0.025	0.091	1.07
	T678A	0.334 ± 0.015	0.107	1.26
	Q737L	0.355 ± 0.023	0.128	1.51
	M844L	0.325 ± 0.010	0.098	1.15
	M844A	0.339 ± 0.015	0.112	1.31
	M987T	0.292 ± 0.019	0.065	0.77
	V1028M	0.304 ± 0.008	0.077	0.91
α-pinene	none	0.282 ± 0.018	-	-
	Wild-type	0.297 ± 0.014	0.015	1.00
	1G1	0.342 ± 0.015	0.060	4.00
	1G2	0.328 ± 0.018	0.046	3.07
	N189H	0.322 ± 0.012	0.040	2.69
	L357Q	0.296 ± 0.009	0.014	0.96
	T678S	0.319 ± 0.008	0.037	2.44
	T678A	0.330 ± 0.012	0.048	3.20
	Q737L	0.340 ± 0.008	0.058	3.89
	M844L	0.318 ± 0.008	0.036	2.41
	M844A	0.327 ± 0.019	0.045	3.00
	M987T	0.293 ± 0.005	0.011	0.75
	V1028M	0.297 ± 0.005	0.015	0.97

^a ^Calculated by taking the difference between the hydrocarbon release rate constants of JA300A expressing no AcrB and JA300A expressing variants of AcrB.

### Growth enrichment of mutant libraries and selection based on determination of efflux rates

Eight mutant libraries of *acrB* were generated by mutagenic PCR and cloned into pMW119. Each library consisted of approximately 20,000 variants and had a mean mutation rate of 3.0 per kilobase (standard deviation 1.9). Library enrichment was initially performed using *n-*octane. Each library was transformed into JA300A and grown. The pre-culture containing a pool of *acrB* variants being expressed at basal level was subjected to the growth enrichment condition as described for *n-*octane. After two additional rounds of sub-cultivation for enrichment, the plasmid mixture for each library was isolated (Figure 3b).

To facilitate rapid selection of improved AcrB from the enriched libraries in the absence of a high-throughput method for measuring efflux rate of *n-*octane, the enriched libraries were preliminarily assayed as variant mixtures to identify the pools with AcrB mutants that improved extrusion of *n*-octane. Discrete clones could then be isolated from these pools for further assay and final selection. Thus, the plasmids of the eight enriched libraries were transformed into JA300A to determine the average rate of *n-*octane efflux for each pool of AcrB variant mixture.

However, the rates of *n-*octane release for the libraries were observed to be similar to that of wild-type AcrB. Sequencing of five discrete clones isolated from a library revealed mutations in the *lac* operator in four clones, even though this region was not subjected to random PCR mutagenesis. This could have arisen because AcrB variants had to be expressed only at basal level for growth selection to maintain sensitivity to *n*-octane such that growth improvement of cells with superior AcrB mutants would be apparent. Consequently, clones with spontaneous mutations in the *lac* operator would have conferred constitutive expression of AcrB, thus providing increased tolerance of *n-*octane and growth advantage over cells expressing AcrB at basal level under the regulation of wild-type *lac* operator. As a result, the libraries were dominated by plasmids with mutant *lac* operator expressing AcrB variants with average performance rather than AcrB mutants with improved efflux efficiency expressed from wild-type *lac* operator. Clearly, *n-*octane could not apply sufficient selection pressure to enable differentiation of variants with good performance from the average ones. To overcome this limitation, we directed our effort towards growth enrichment using 1-octanol.

In contrast to selection conditions with *n-*octane, AcrB expression was induced with IPTG before and during growth enrichment with 1-octanol to overcome the higher toxicity to *E. coli* compared to *n-*octane. After two rounds of sub-cultivation, the enriched plasmid mixture was purified and the variants were assayed for *n-*octane release rate. Two of the eight enriched libraries contained variants with enhanced *n-*octane efflux efficiency. Five discrete clones were isolated from each of the two libraries. Sequencing of the *acrB* mutants identified only one variant from each library and no mutation was found in the *lac* operator for both variants. This result indicates that the selection pressure from the growth enrichment conditions effectively eliminated less tolerant cells harboring less efficient AcrB mutants. These two AcrB variants are hereafter designated 1G1 and 1G2. The plasmids containing the genes are named p1G1 and p1G2, respectively. The mutations in 1G1 are N189H and T678S, and those in 1G2 are L357Q, Q737L, M844L, M987T, V1028M.

### Characterization of AcrB variants and structural analysis

**Figure 5:**
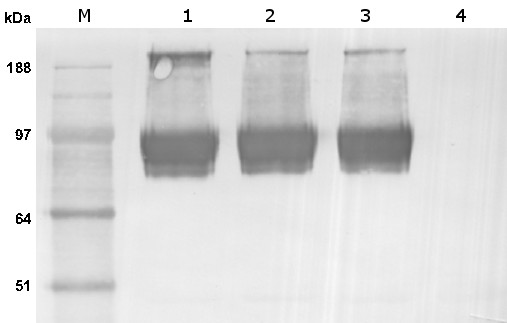
**Expression levels of AcrB variants. **M, marker; lane 1, wild-type AcrB; lane 2, 1G1; lane 3, 1G2; lane 4, negative control (pMW119).

JA300A harboring p1G1 and p1G2 were cultivated from single colonies and induced with IPTG to express the AcrB mutants. The growth rates of the cells in the presence of 1-octanol and the efficiency of the AcrB variants in effluxing *n*-octane were investigated. Both 1G1- and 1G2-expressing JA300A showed significantly faster growth rates in the presence of 1-octanol, reaching OD_600_ of 2.90 and 1.48, respectively, compared to 1.05 of wild-type AcrB after 48 h of growth (Figure 1b). The variants also exhibited over 40% increase in *n*-octane efflux rate relative to wild-type AcrB (Table 1). To examine the possibility that improvements in performance were due to increased AcrB expression of the mutants, western blotting was performed to compare the AcrB expression levels between the variants. Constructs of pAcrB, p1G1 and p1G2 with hexahistidine-tags inserted at the C-termini were synthesized, and the proteins expressed with IPTG induction were probed using anti-hexahistidine antibodies. There was negligible difference in the expression levels of 1G1 and 1G2 compared to wild-type AcrB (Figure 5), indicating that the increased efflux of *n-*octane and enhanced tolerance of the cells expressing 1G1 and 1G2 were due to improved performance of the mutant proteins. Thus, we have demonstrated successful isolation of AcrB mutants with improved *n-*octane efflux efficiency from a competitive growth selection based on 1-octanol tolerance.

Single-site mutants of the seven mutations identified in 1G1 and 1G2 were generated to investigate their individual effects on *n*-octane efflux (Table 1). L357Q, M987T, V1028M showed reduced rate of *n*-octane extrusion. Q737L and M844L increased the rate of *n*-octane efflux by 51 and 15%, respectively. Interestingly, N189H and T678S improved efflux of n-octane only slightly (less than 10%) although 1G1, which possess these two mutations, exhibited 47% faster efflux of *n*-octane. These results suggest that N189H and T678S may work synergistically.

**Figure 6:**
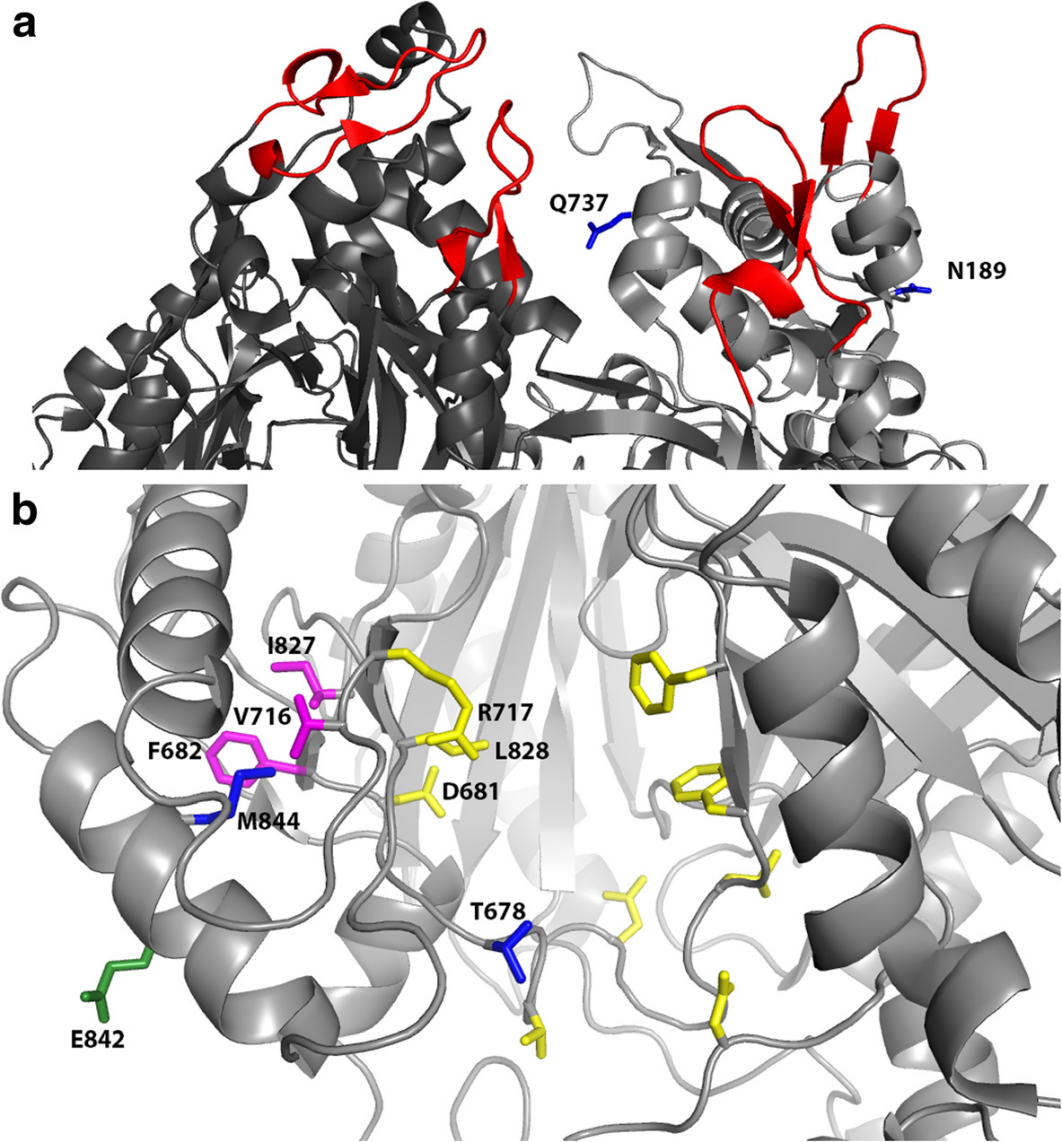
**Structural analysis of the mutations at N189, T678, Q737 and M844 (PDB ID: 2DHH).** The “Access” conformation of the structure was used. In (**a**), N189 and Q737 (in blue) are near the hairpins (in red) that interact with TolC to form the AcrB-TolC complex. Q737 is in the vicinity of a hairpin on the adjacent protomer (in dark gray). In (**b**), T678 and M844 are shown in blue. Residues that were reported to be in the substrate path leading to the binding pocket are colored yellow [24,25]. The amino acids F682, V716 and I827 (shown in pink) are in close proximity to M844 and flank residues in the substrate path (D681, R717 and L828). M844 is also on the same α-helix as E842 (shown in green) which is part of the vestibule leading to the binding pocket.

To gain structural-functional insights of the seven mutations in 1G1 and 1G2, the positions of the amino acid substitutions were located on a known asymmetric structure of AcrB (PDB ID: 2DHH) [21]. Analysis of the structure shows that L357Q, M987T, V1028M were in the transmembrane domain at locations unlikely to contribute to substrate efflux, consistent with the lowered *n*-octane efflux rates of the single-site mutants relative to wild-type AcrB. An overview of the positions of N189, T678, Q737 and M844 on the structure of AcrB is illustrated in Figure 6 using the protomer with the “Access” conformation. N189H and Q737L are located near the hairpins in the TolC docking domains (Figure 6a), which are responsible for interaction with TolC to form an AcrB-TolC complex [22]. This interaction is important to facilitate transfer of substrates from AcrB to TolC for exportation out of the cell via the TolC channel. The exact mechanism how N189H and Q737L promotes efflux of *n-*octane is unclear. However, the proximity of the two mutations to the hairpins in the TolC docking domains suggests they might assist the assembly of the AcrB-TolC complex. Particularly, Q737 is in the vicinity of a hairpin on the adjacent protomer, thus the shortening of the side chain due to Q737L might stabilize or improve the assembly or interactions between AcrB and TolC for efficient export of *n*-octane.

T678S and M844L occur close to each other along two proposed transport paths i.e. the vestibule between protomers and the cleft in the periplasmic domain (Figure 6b) [23,24]. Analysis of the structure of AcrB, which shows functional rotation of the protomers, revealed that T678 is near the entrance at the lower cleft in the periplasmic domain leading to the binding pocket of AcrB and exhibits large displacement when transitioning between the “Access”, “Binding” and “Extrusion” conformations (Additional file 1: Figure S2) [21]. In the “Extrusion” conformation, T678, which is in the PC2 subdomain, is extremely close to N667 (2.6 Ǻ) in the PC1 subdomain as the entrance is closed off. The distance between T678 and N667 increases as the entrance opens to allow substrate access into AcrB when the conformation transitions to “Access” and “Binding”, before closing off the entrance again to assume the “Extrusion” conformation. Thus, we propose that shortening the side chain of threonine in the T678S mutant would likely widen the entrance at the lower cleft in the periplasmic domain and permit hydrocarbons more access time into the binding pocket for extrusion. The role of M844L is less obvious. M844 is on the same α-helix as E842 that has been identified as one of the residues lining the vestibule leading to the binding pocket [23], thus mutation to a leucine might cause a conformational change that facilitates hydrocarbon entry into the transport channel through the vestibule. Additionally, the side chain of M844 is pointing in the opposite direction of the vestibule towards adjacent loops bearing D681, R717, L828 that line another channel to the binding pocket in the cleft [23-25]. In fact, it is in close proximity to F682, V716 and I827 (3.9-4.3Ǻ) that flank D681, R717 and L828, respectively (Figure 6b). Incidentally, M844 is located in the PC2 subdomain of AcrB in a region that exhibits large conformational changes during the displacement of the drug doxorubicin [26]. It is possible that the shorter side chain of leucine causes distal conformational changes that re-position D681, R717 and L828 such that the channel leading to the binding pocket is enlarged or the dynamics of substrate efflux is altered. This phenomenon has been observed in other proteins [27,28]. Crystal structures would be required to validate these hypotheses. Nevertheless, these propositions on the roles of the T678S and M844L mutations are supported by the increased *n-*octane efflux by the T678A and M844A variants (Table 1), which have the side chains shortened to a methyl group. The effect of T678A is also consistent with the observation that increasing the bulk of residue A677, which is adjacent to T678, reduced drug efflux [25].

**Figure 7:**
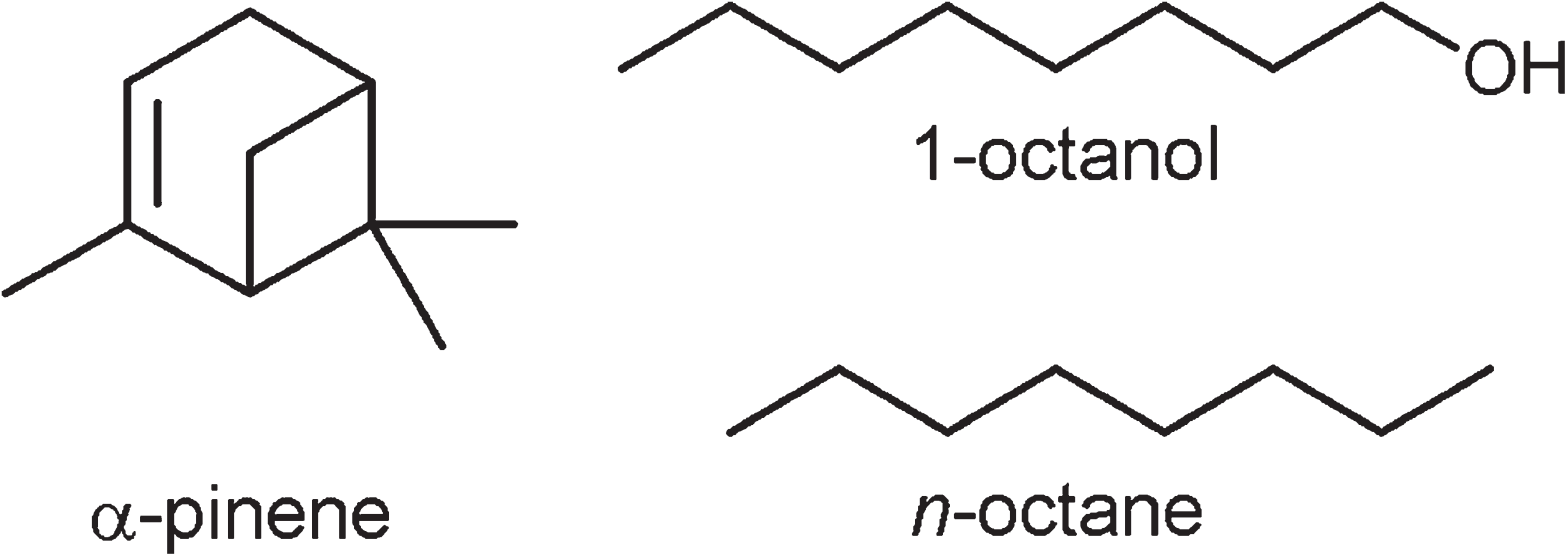
Structures of hydrocarbons used in this work.

To investigate the ability of 1G1, 1G2 and the single-site mutants in effluxing biofuels that are structurally dissimilar to 1-octanol and *n*-octane, which are conformationally flexible linear compounds, we chose a cyclic and conformationally more rigid biofuel candidate, α-pinene, as the test substrate. The rate of α-pinene release was assayed (Table 1) and interestingly, the mutants that improved efflux of *n*-octane also enhanced the efflux rate of α-pinene (Table 1) despite the structural dissimilarity of the substrates (Figure 7). This suggests that the beneficial effects of the mutations are not specific to 1-octanol and *n-*octane only, but may be generally important for improving efflux of small hydrophobic hydrocarbon molecules.

Three known antibiotic substrates of AcrB [29] of varying molecular weight – nalidixic acid (232.24 g mol^-1^), chloramphenicol (323.13 g mol^-1^) and tetracycline (444.44 g mol^-1^) – were chosen to investigate the antibiotic resistance of the mutants that improved *n*-octane efflux (i.e. 1G1, 1G2, N189H, T678S, T678A, Q737L, M844L and M844A). Interestingly, none of the mutants showed increased antibiotic resistance towards the antibiotics. JA300A expressing wild-type and AcrB mutants have minimum inhibitory concentrations (MICs) of 5.0, 5.0 and 1.0 mg/L for chloramphenicol, nalidixic acid and tetracycline, respectively. The MICs for the control strain (JA300A with pMW119) towards chloramphenicol, nalidixic acid and tetracycline are 1.5, 1.5 and 0.1 mg/L, respectively. The neutral effect of the mutations towards antibiotic resistance could be because the mechanisms for extrusion of antibiotics and small hydrophobic molecules are different. For example, substrate recognition through interactions between amino acids in the transport pathway of AcrB and the polar functional groups in antibiotics is required for antibiotic efflux [21] whereas such interactions are absent with small hydrophobic molecules. Nevertheless, it is advantageous that the mutations beneficial for *n*-octane efflux do not increase antibiotic resistance because that would eliminate the possibility of generating pathogenic bacteria with high antibiotic resistance. This would be particularly important if strains with the mutant AcrBs were used in large volumes for mass production of hydrocarbons.

## Conclusions

A ‘competitive growth’-based selection strategy was developed in this study for rapid isolation of AcrB mutants with improved efflux efficiency of *n-*octane. Although the crystal structure and substrate path of AcrB are known, the mechanism of AcrB involves a highly dynamic peristaltic action and functional rotation between the protomers [21]. Therefore, we chose directed evolution over rational design to engineer AcrB because it could enable discovery of variants with mutations that would not have been obvious enough to be identified. Indeed, with the exception of T678S which lies in the substrate path, the other amino acid changes that benefit *n*-octane efflux, i.e. N189H, Q737L and M844L, are distant from the substrate path. We reasoned that these mutations may improve substrate efflux by enlarging the substrate path, altering the substrate efflux dynamics and facilitating the assembly of the AcrB-TolC complex. These mutations are also beneficial for effluxing other small hydrophobic molecules, as illustrated by the increased extrusion rate of α-pinene.

We successfully engineered AcrB for enhanced efflux efficiency of *n-*octane by competitive growth selection for tolerance towards the more toxic substrate surrogate, 1-octanol. This strategy of employing a toxic substrate surrogate for competitive growth enrichment is particularly beneficial for engineering efflux pumps to accelerate extrusion of biofuel with low toxicity, such as *n*-octane and higher alkanes [8], since mildly toxic compounds could not select for AcrB mutants effectively for enhanced efflux. These improved AcrB can now be used to improve the yield of biofuel production and/or increase cell tolerance to the synthesized hydrocarbon [9]. The next step forward would be to validate the use of these mutant pumps in engineered bacterial systems that biosynthesize small biofuel molecules such as *n-*octane and α-pinene in vivo. Much efforts are currently focused on metabolic engineering activities for biofuel production in microbes [1-6] and we expect the outcome of this work to underpin the development of scalable microbial platforms for biofuels production.

## Methods

### Strains, plasmids, oligonucleotides, chemicals and culture media

*E. coli* XL10-Gold {(δ(*mcrA*)*183* δ(*mcrCB-hsdSMR-mrr*)*173 endA1 supE44 thi-1 recA1 gyrA96 relA1 lac* Hte [F’ *proAB lacI*^q^*Z* δ*15* Tn*10* (Tet^r^) Tn*5* (Kan^r^) Amy]} (Stratagene) was the host strain used for plasmid construction. JW0451-2 [F^-^, *δ(araD-araB)567*, *δlacZ4787*(::rrnB-3), *δacrB747::kan*, *λ*^*-*^, *rph-1*, *δ(rhaD-rhaB)568*, *hsdR514*] strain and P1vir phage were purchased from The Coli Genetic Stock Center (Yale University, USA). JA300 (F^-^*thr leuB6 trpC1117 thi rpsL20 hsdS*) strain was obtained from ATCC. Its *acrB*-inactivated derivative, JA300A, was generated by P1vir phage transduction [30] with JW0451-2. JA300A was used for library enrichment, hydrocarbon resistance studies and efflux assays. *acrB* gene was cloned from the *E. coli* strain W3110 [F^-^ IN(*rrnD-rrnE*)*1*]. The low-copy-number vector pMW119 (WAKO Pure Chemical Industries, Osaka, Japan) was used for cloning and expression under the control of the *lac* promoter. Oligonucleotides were synthesized by 1^st^ Base (Singapore) and the sequences are shown in Additional file 1: Table S1. All chemicals were purchased from Sigma-Aldrich (Singapore) and of purity higher than 98%.

Lysogeny broth (LB) consisting of 1% tryptone, 0.5% yeast extract and 1% NaCl was used for cell cultivation. Where appropriate, ampicillin (100 μg/mL) and/or IPTG (0.1 mM) were added to the medium. Hereafter, LBA and LBI refers to LB medium supplemented with ampicillin and IPTG, respectively, and LBAI refers to LB medium with both ampicillin and IPTG added.

### DNA manipulation

Standard techniques were used for DNA manipulation, including isolation and purification of plasmids and DNA fragments, agarose gel electrophoresis, restriction enzyme digestion, ligation and transformation of *E. coli*[31]. All restriction enzymes and DNA-modifying enzymes were purchased from Fermentas (Singapore).

### Cloning of *acrB*

*acrB* was amplified from W3110 chromosomal DNA by PCR using the primers acrB-F and acrBCln-R, which contain the BamHI and SacI restriction sites, respectively. The 3.2-kb PCR fragment was digested with BamHI and SacI and the restricted DNA was cloned into the 4.2-kb vector pMW119 to generate the 7.4-kb plasmid pAcrB. The sequence of the cloned *acrB* was verified by DNA sequencing.

### Study of growth inhibition by *n*-octane and 1-octanol

pMW119 and plasmids of *acrB* variants were transformed into electrocompetent JA300A. Overnight cultures of the strains were diluted in 3 mL LB medium to OD_600_~0.03. To study the growth inhibition of cells by *n-*octane, the diluted cells were shaken at 37°C with 1 mL *n-*octane, both in the presence and absence of 0.1 mM IPTG, and the OD_600_ was measured hourly. The growth inhibition of the strains by 1-octanol was performed similarly with 0.025% and 0.050% (v/v) 1-octanol in LBI medium and OD_600_ absorbance was measured every 24 h. Cultures without *n-*octane and 1-octanol were grown in parallel as controls.

### Competitive growth studies

JA300A was transformed with either pMW119 or pAcrB. Overnight cultures of the two strains were grown separately and mixed in a 1:1 ratio. A 400 μL aliquot of the mixed cells was inoculated into 20 mL of LB medium and split into two 10 mL portions. To one of the portions, 3 mL of *n-*octane was added. Both cultures were grown overnight at 37°C with shaking and subcultivated by 50-fold dilution in the respective growth media. After two more rounds of subcultivating, 200 μL of the final cultures served as inoculum for 10 mL of LBA medium. Plasmids were isolated from the overnight cultures for agarose gel electrophoresis after BamHI digestion.

For competitive growth study with 1-octanol, two 10 mL portions of mixed cells were similarly prepared except LBI medium was used. 1-Octanol was added to one of the portions to a final concentration of 0.05% and both cultures were grown for 2 days at 37°C with shaking. After another round of subcultivating, the cells were diluted 50-fold in LBA medium and grown for plasmid isolation. The plasmids were subsequently digested with BamHI and analyzed by agarose gel electrophoresis.

### Mutant library construction

Mutagenic PCR was performed to introduce random mutations into the *acrB* gene. Eight 50 μL PCR reaction mixtures were prepared and handled individually. Each reaction consisted of 10 ng of pAcrB as template, 0.2 μM of each primer acrB-F and acrB-R, 0.2 mM dATP, 0.2 mM dGTP, 1 mM dCTP, 1 mM dTTP, 10 mM Tris–HCl (pH 8.8), 50 mM KCl, 0.08% (v/v) Nonidet P40, 7 mM MgCl_2_, 1 μL DMSO, 5 U Taq DNA polymerase (Fermentas). The thermocycling program used consisted of an initial denaturation at 94°C for 2 min, followed by 20 cycles of denaturation at 94°C for 10 s and concurrent annealing and extension at 72°C for 210 s. The PCR products were restricted with BamHI and SacI and cloned into pMW119, effectively generating eight mutant libraries. The ligated products were recovered by transformation into electrocompetent XL-10 Gold and isolation of the plasmid mixtures from cells cultured in LBA medium. Mutation rates of the libraries were determined by plating 10 μL of the initial transformants on LBA-agar plates and sequencing plasmids isolated from 10 random single colonies picked from the plates.

### Competitive growth enrichment of mutant libraries with *n*-octane and 1-octanol

The purified mutant library plasmid mixtures were transformed into electrocompetent JA300A. After 1 h of outgrowth, a pre-culture was prepared by growing the transformants overnight in 10 mL of LBA medium at 37°C with continuous shaking.

For growth enrichment with *n-*octane, a 200 μL aliquot of the pre-culture was inoculated into 10 mL of LB medium saturated with a 3-mL overlay of *n-*octane, and shaken overnight at 37°C. The cultures were enriched by two more rounds of sub-culturing.

For growth enrichment with 1-octanol, a 200 μL aliquot of culture was inoculated into 10 mL of LBAI medium. The cells were grown overnight with shaking at 37°C to induce expression of AcrB. The overnight culture served as inoculum for 10 mL of LBI medium with 0.05% of 1-octanol. This culture was grown at 37°C with shaking for 2 days. The cultures were subcultivated once more for enrichment.

To isolate plasmid DNA from the enriched libraries, a 200 μL aliquot of the final enrichment culture was inoculated into 10 mL of LBA medium and the cells were grown overnight at 37°C for miniprep. The enriched library DNA was transformed into XL-10 Gold and plated on LBA agar. Plasmids of discrete clones were isolated from cultures grown from single colonies picked from the plate of cells and sequenced (Figure 3b).

### Assay for rate of hydrocarbon release from *E. coli*

The rate of hydrocarbon release from *E. coli* cells were determined by adapting published methods [8,16]. Pre-cultures of JA300A cells transformed with various plasmids were prepared. For preliminary assays of enriched *acrB* libraries, the pre-culture was prepared by transformation of the mixture of plasmids into electrocompetent JA300A cells and growing the cells overnight in 10 mL LBA medium after 1 h of outgrowth. For assays of individual variants, cells for the pre-cultures were grown from a single colony of JA300A transformed with the plasmid picked from an LBA agar plate.

The pre-cultures were diluted 50-fold with 10 mL LBAI medium and grown for 16 h at 37°C. The cells were washed thrice by centrifugation (2000g, 3 min) and resuspended in 10 mL PPB (20 mM sodium phosphate pH 7 and 1mM MgCl_2_). After another centrifugation cycle to pellet the cells, the cells were resuspended in 10 mL PPB at OD_600_~1.5. Carbonyl cyanide 3-chlorophenylhydrazone (10 mM stock solution in DMSO) was added to the cells to a final concentration of 10 μM and the cell suspensions were shaken at 160 rpm for 15 min at 37°C. *n-*Octane or α-pinene (0.5 mL) was added to each sample and the biphasic mixture was further shaken at 200 rpm for 1 h at 37°C. The cells were then pelleted at 2000g for 3 min. The supernatant and hydrocarbon were carefully and thoroughly aspirated. The cells were resuspended in 10 mL PPB with 50 mM glucose, then incubated at 37°C and shaken at 160 rpm. One milliliter aliquots were transferred into microcentrifuge tubes in duplicate periodically (0-7min). The cell aliquots were immediately centrifuged (21000 g, 1 min) and the supernatant was removed. The cell pellets were resuspended in 500 μL dH_2_O before extraction with 750 μL CHCl_3_ for 3 h. The organic extracts were removed and analyzed by gas chromatography-mass selective detector (GC-MS). (Figure 3a).

### Site-directed and insertional mutagenesis

Single-site N189H, T678A, Q737L and M844A *acrB* mutants were generated from pAcrB using the QuikChange (Stratagene) protocol for site-directed mutagenesis. The primers used were as follow: N189H-F, N189H-R for N189H *acrB*; T678A-F, T678A-R for T678A *acrB*; Q737L-F, Q737L-R for Q737L *acrB*; M844A-F, M844A-R for M844A *acrB*. The PCR reaction mixtures consisted of 20 ng pAcrB, 200 μM dNTP, 1.75 mM MgSO_4_, 1 μL DMSO, 1 U KOD Hot Start DNA polymerase (Novagen), 0.3 μM mutagenic primers and 5 μL 10x KOD Hot Start DNA polymerase PCR buffer made up to 50 μL with deionized water. The reaction mixtures were subjected to an initial denaturation at 95°C for 2 min, followed by 20 cycles of denaturation at 95°C for 20 s, annealing at 60°C for 20 s and extension at 70°C for 240 s.

Hexahistidine-tags were added to the C-termini of *acrB* genes by appending four histidines to the two that are naturally present in protein via insertional mutagenesis [32]. PCR mixtures consisting of 20 ng plasmid templates, 200 μM dNTP, 1.75 mM MgSO_4_, 1 μL DMSO, 1 U KOD Hot Start DNA polymerase (Novagen), 0.2 μM of mutagenic primers acrBHis-F and acrBHis-R each and 5 μL 10x KOD Hot Start DNA polymerase PCR buffer made up to 50 μL with deionized water were prepared. The reaction mixtures were subjected to an initial denaturation at 95°C for 2 min, followed by 15 cycles of denaturation at 95°C for 20 s, annealing at 50°C for 20 s and extension at 70°C for 210 s.

To isolate plasmids of the mutants, the PCR mixtures were digested with *Dpn*I at 37°C for 3 h and transformed into electrocompetent XL-10 Gold cells. The cells were plated onto LBA agar and incubated at 37°C overnight. Cells were grown from single colonies and plasmids were isolated by miniprep kit. Mutations were verified by sequencing.

### Immunoblot analysis

Cells expressing hexahistidine-tagged AcrB were cultivated as described for assaying the rate of hydrocarbon release. The OD_600_ of the cultures were adjusted to 1.0, and 1-mL aliquots of the cells were centrifuged (21000g, 2 min). The cell pellets were resuspended in a buffer containing 20 mM Tris–HCl (pH 8.0), 5 mM EDTA, 8 M urea, 10 mM phenylmethanesulfonyl fluoride and 2% sodium dodecyl sulfate, and shaken gently at room temperature for 1 h to lyse the cells and solubilize AcrB. The lysates were mixed with an equal volume of 2x loading dye (Sigma-Aldrich) and incubated at 37°C for 10 min before being analyzed by SDS-PAGE. The proteins were transferred to polyvinylidene fluoride membranes (Bio-Rad) electrophoretically and probed using anti-hexahistidine antibody conjugated with horse radish peroxidase. The hexahisitidine-tagged AcrBs were detected colorimetrically on the membrane using 3,3’,5,5’-tetramethylbenzidine.

### Antibiotic resistance assay

JA300A harboring pMW119 and plasmids with genes for wild-type, 1G1, 1G2, N189H, T678S, T678A, Q737L, M844L and M844A *acrB* were grown overnight in LBA at 37°C with shaking at 200 rpm. Approximately 10^3^ cells were added to LB with various concentrations of chloramphenicol (0.5-10 mg/L), nalidixic acid (0.5-10 mg/L) and tetracycline (0.05 - 3mg/L) in 96-well microtitre plates and grown at 37°C with shaking at 200 rpm for 20 h. The MIC is the lowest antibiotic concentration that inhibited growth. Triplicated experiments gave consistent MIC.

### Structural analysis

The locations of the mutations were studied on a published structure of AcrB (PDB ID: 2DHH). Rendered images of protein structures were generated with Pymol [33].

## Abbreviations

IPTG: Isopropyl-β-D-thiogalactopyranoside; CCCP: Carbonyl cyanide 3-chlorophenylhydrazone; PCR: Polymerase chain reaction; LB: Lysogeny broth; LBA: LB supplemented with ampicillin; LBI: LB supplemented with IPTG; LBAI: LB supplemented with ampicillin and IPTG; DMSO: Dimethyl sulfoxide; GC-MS: Gas chromatography-mass selective detector.

## Competing interests

The authors declare that they have no competing interests.

## Authors’ contributions

JLF and SSJL designed the study and JLF performed the experimental work. JLF and SSJL co-wrote the manuscript. Both authors read and approved the final manuscript.

## Supplementary Material

Additional file 1: Table S1 Sequences of oligonucleotides used in this work. **Figure S1**. Effect of 100 μM IPTG on the growth of JA300A/pMW119 (triangle) and JA300A/pAcrB (circle). The growth of the strains were not appreciably affected by the presence (filled symbols, solid lines) and absence (open symbols, dashed lines) of 100 100 μM IPTG. **Figure S2**. Positions of T678 in different conformations of AcrB (PDB ID: 2DHH). As AcrB cycles through the (A) “Extrusion”, (B) “Access” and (C) “Binding” conformations during functional rotation, the position of T678 varies significantly. T678 is extremely close to N667 in the PC1 domain in the “Extrusion” conformation but the distance between the residues increases in the “Access” and “Binding” conformation to allow substrate entry into AcrB. Click here for file
