# Supplementary material for: Directed evolution of an E. coli inner membrane transporter for improved efflux of biofuel molecules
Source: Biotechnol Biofuels. 2013 May 21;6:81. doi: 10.1186/1754-6834-6-81 (PMC3680313; doi:10.1186/1754-6834-6-81)
Supplement: Additional file 1: Table S1 — Sequences of oligonucleotides used in this work. Figure S1. Effect of 100 μM IPTG on the growth of JA300A/pMW119 (triangle) and JA300A/pAcrB (circle). The growth of the strains were not appreciably affected by the presence (filled symbols, solid lines) and absence (open symbols, dashed lines) of 100 100 μM IPTG. Figure S2. Positions of T678 in different conformations of AcrB (PDB ID: 2DHH). As AcrB cycles through the (A) “Extrusion”, (B) “Access” and (C) “Binding” conformations during functional rotation, the position of T678 varies significantly. T678 is extremely close to N667 in the PC1 domain in the “Extrusion” conformation but the distance between the residues increases in the “Access” and “Binding” conformation to allow substrate entry into AcrB. [file 1754-6834-6-81-S1.docx]

**Supplementary Table**

Table S1. Sequences of oligonucleotides used in this work.

| Name | Sequence |
| --- | --- |
| acrBCln-R | GGCCAGTGAGCTCTTATCAATGATGATCGACAGTAT |
| acrB-F | TCTAGAGGATCCCTAACTTAAACAGGAGCCGTTAAGACATG |
| acrB-R | GACGTTGTAAAACGACGGCCAGTGAATTCGAGCTCTTATCA |
| acrBHis-F | TGATAAGAGCTCGAATTCACTGG |
| acrBHis-R | ATGGTGATGGTGATGATGATCGACAGTATGGCTGT |
| T678A-F | GAACTGGGTACTGCAGCGGGCTTTGACTTTGAG |
| T678A-R | CTCAAAGTCAAAGCCCGCTGCAGTACCCAGTTC |
| M844A-F | TGAAGCAATGGAGCTGGCGGAACAACTGGCGAG |
| M844A-R | CTCGCCAGTTGTTCCGCCAGCTCCATTGCTTCA |
| F664A-F | AAGATGCGATGGTTGCGGCCTTTAACCTGC |
| F664A-R | GCAGGTTAAAGGCCGCAACCATCGCATCTT |
| N189H-F | TGCGTATCTGGATGCACCCGAATGAGCTGA |
| N189H-R | TCAGCTCATTCGGGTGCATCCAGATACGCA |
| Q737L-F | ACCAGGAAAAAGCGCTGGCGCTGGGTGTTTC |
| Q737L-R | GAAACACCCAGCGCCAGCGCTTTTTCCTGGT |

**Supplementary Figure**


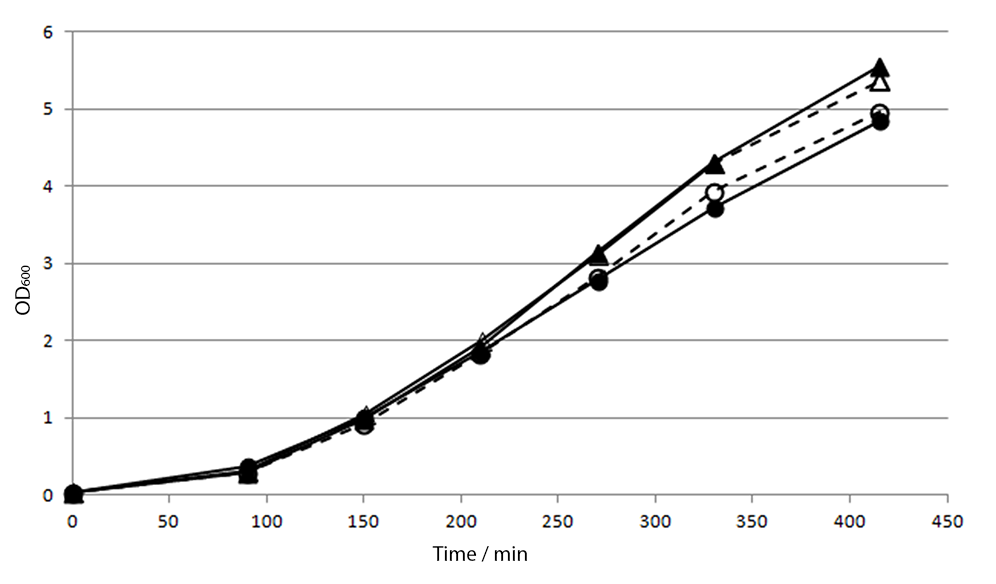


Figure S1. Effect of 100 μM IPTG on the growth of JA300A/pMW119 (triangle) and JA300A/pAcrB (circle). The growth of the strains were not appreciably affected by the presence (filled symbols, solid lines) and absence (open symbols, dashed lines) of 100 100 μM IPTG.


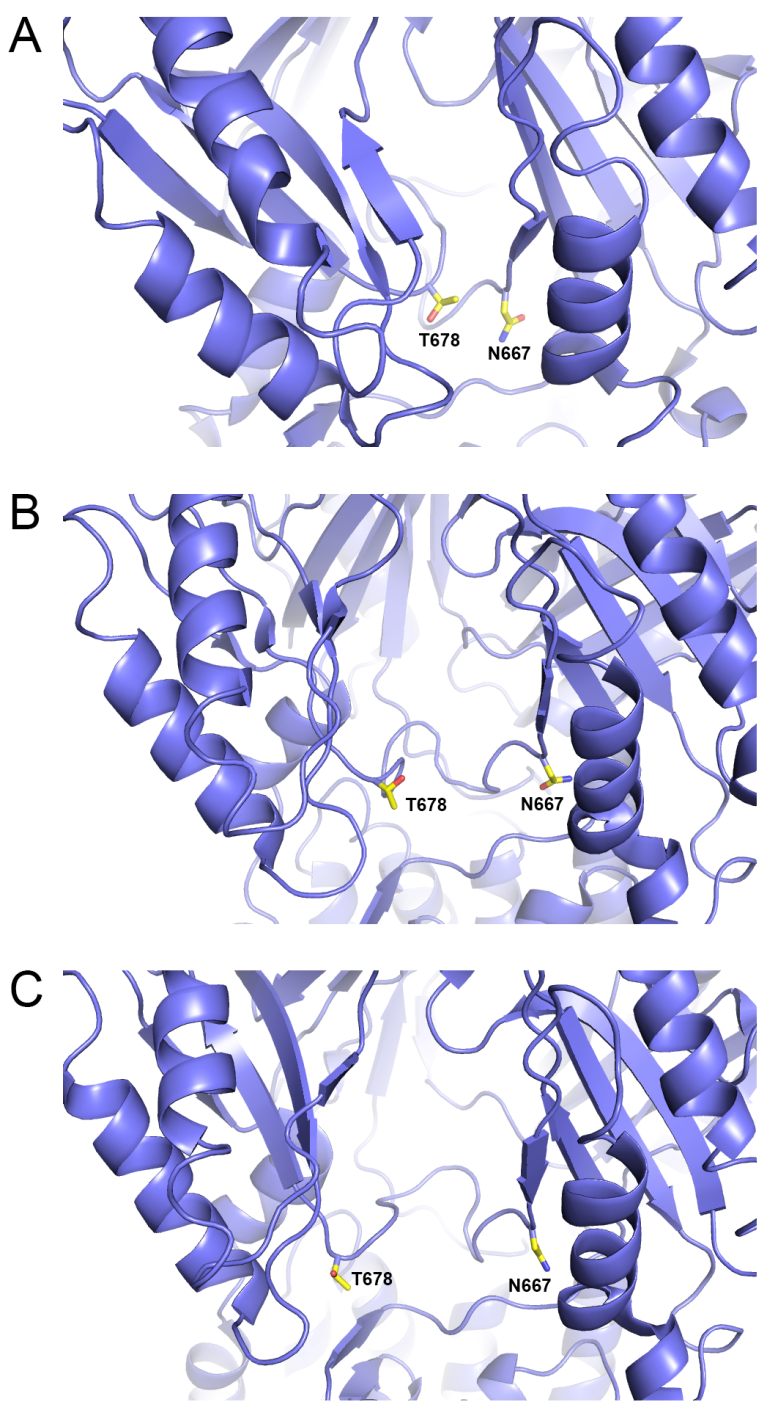


Figure S2. Positions of T678 in different conformations of AcrB (PDB ID: 2DHH). As AcrB cycles through the (A) “Extrusion”, (B) “Access” and (C) “Binding” conformations during functional rotation, the position of T678 varies significantly. T678 is extremely close to N667 in the PC1 domain in the “Extrusion” conformation but the distance between the residues increases in the “Access” and “Binding” conformation to allow substrate entry into AcrB.
